# Ptaquiloside-induced cytotoxicity in Crandall feline kidney and HGC-27 cells

**DOI:** 10.3892/ol.2014.2378

**Published:** 2014-07-24

**Authors:** BEGUM YURDAKOK, GORKEM KISMALI, DOGUKAN OZEN

**Affiliations:** 1 Department of Pharmacology and Toxicology, Ankara University Faculty of Veterinary Medicine, Ankara 06110, Turkey; 2 Department of Biochemistry, Ankara University Faculty of Veterinary Medicine, Ankara 06110, Turkey; 3 Department of Biostatistics, Ankara University Faculty of Veterinary Medicine, Ankara 06110, Turkey

**Keywords:** cytotoxicity, ptaquiloside, Crandall feline kidney cells, HGC-27 cells

## Abstract

Ptaquiloside (PTA) is a potent genotoxic carcinogenic compound, which is found in bracken ferns and predominantly causes gastric tumors in humans, as well as bladder tumors and chronic enzootic hematuria in cattle. The underlying molecular mechanisms of PTA remain a topic for interdisciplinary investigation. The aim of the present study was to determine the possible cytotoxic effect of 24 h of PTA exposure in Crandall feline kidney (CrFK) and human gastric cells (the HGC-27 cell line) using the 3-(4,5-dimethylthiazol-2-yl)-2,5-diphenyltetrazolium bromide (MTT) assay and lactose dehydrogenase (LDH) analysis. The cytotoxic effects of PTA (0.0005–500 μg/ml) were found to increase in a dose-dependent manner, whereby the half maximal inhibitory concentration values were 11.17 and 11.86 μg/ml in the CrFK cells, and 2.03 and 2.56 μg/ml in the HGC-27 cells, by LDH and MTT assay, respectively. The results of the present study are consistent with those of previous studies associated with the cytotoxicity of PTA; however, cytotoxicity was identified to occur at significantly lower doses. This cytotoxic effect *in vitro* at particularly high doses may be linked to the initiation of carcinogenesis as a result of oxidative stress.

## Introduction

In Turkey, different types of fern, including *Pteridium aquilinum* (L.) Kuhn (a poisonous plant, termed bracken fern, which is grown in the feeding grounds of the Black Sea and the Marmara Region) are consumed by water buffaloes and cattle, resulting in chronic enzootic hematuria (CEH). This is a chronic disease, which is economically significant worldwide (1).

*P. aquilinum* (L.) Kuhn causes numerous pathological changes, as it contains a variety of toxic substances that are carcinogenic, clastogenic and mutagenic, including a radiomimetic carcinogenic compound, norsesquiterpene ptaquiloside (PTA), thiaminase type I, braxin A, B, and C and quercetin, which is known to be a powerful agent responsible for DNA alteration (2,3).

At sublethal or subtoxic levels, PTA causes genotoxicity via the formation of numerous DNA adducts, with the predominant labile adducts occurring at the N-3 position of adenine, and, to a minor extent, at the N-7 position of guanine (4). *H-Ras* activation has also been demonstrated to be an early event of PTA-induced carcinogenesis in a rat model (5). The overexpression of this protein has been demonstrated in bladder neoplasia and cystitis in animals that were naturally exposed to bracken fern. In addition, cyclin D1 and p53 overexpression has been observed in endothelial-derived bovine urinary bladder tumors. Furthermore, cyclin D1 overexpression was found to positively correlate with a high tumor grade and occurred in 53% of hemangiomas, 82% of hemangioendotheliomas and 95% of hemangiosarcomas (5). PTA-induced carcinogenesis activates various cellular specific cellular signaling pathways in animals exhibiting bovine enzootic hematuria (6).

The bracken carcinogen, PTA, may also be transferred to humans directly when milk, obtained from bracken-exposed animals, or the underground water (contaminated by leaching from the plant) is consumed. Epidemiological studies have revealed that individuals consuming these products in bracken-infested areas exhibit a higher risk of cancer (2,11). Milk from cows that have consumed bracken containing high concentrations of PTA may be hazardous to humans, which was demonstrated by previous studies on rats, which investigated milk consumption from bracken-fed cows (7,8). Depending on the period during which the plant is consumed and the quantity that is ingested, the radiomimetic principle is responsible for three different clinicopathological conditions, which are predominantly observed in cattle; hemorrhagic diathesis, CEH and carcinomas of the upper digestive tract. The nature of bladder tumors, which are associated with the ingestion of *P. aquilinum*, are particularly unusual and mixed tumors (epithelial and mesenchymal in origin) have previously been described (6).

The multifactorial origin of gastric cancer includes environmental factors that are predominantly associated with diet (9). The increasing awareness concerning the risk exhibited by continuous exposure to carcinogenic substances in food has prompted studies, which investigate the possibility that PTA may present a food pollutant that is derived from cattle (10). For example, individuals who were raised in bracken-infested areas and consumed the infested buttermilk (a potential vector for bracken carcinogens) were identified to exhibit an increased risk of gastric cancer (11).

There are a small number of studies regarding the cytotoxic effects of PTA, or fern extracts containing PTA, in various cell lines (12–14), such as AGS and MKN-45 (9), whereby genotoxic activity and activation of DNA damage responses were recorded. However, the association between tumor proliferation and the cytotoxicity of these compounds at varying doses remains unclear. Therefore, the aim of the present study was to determine the possible cytotoxic effects of PTA on Crandall feline kidney (CrFK) cells, derived from the kidney tissue of a normal domestic kitten, and human gastric cells (the HGC-27 cell line), derived from the metastatic lymph node of a gastric cancer patient diagnosed histologically with undifferentiated carcinoma. The 3-(4,5-dimethylthiazol-2-yl)-2,5-diphenyltatrazolim (MTT) and lactose dehydrogenase (LDH) assays were performed to determine the percentage cytotoxicity.

## Materials and methods

### Cell culture and treatment

The CrFK cell line was provided by Ankara University Faculty of Veterinary Medicine (Ankara, Turkey) and the HGC-27 cell lines were provided by Padova University (Padova, Italy). The cell culture medium used for the HGC-27 cell lines was Eagle’s minimal essential medium (Gibco-BRL, Carlsbad, CA, USA) supplemented with 10% heat-inactivated fetal bovine serum (FBS) (26140-079; Invitrogen Life Technologies, Carlsbad, CA, USA), 1% nonessential amino acid solution (11140076; Invitrogen Life Technologies), 2 mM L-glutamine (Invitrogen Life Technologies) and 1% antibiotic-antimycotic solution (Sigma-Aldrich, St. Louis, MO, USA). CrFK cells were cultured in Dulbecco’s modified Eagle’s medium (Sigma-Aldrich) with 10% FBS and 1% antibiotic-antimycotic solution. The antibiotic-antimycotic solution (100X; 15240-112; Invitrogen Life Technologies) contained 10,000 units of penicillin G, 10,000 μg streptomycin sulfate and 25 μg/ml amphotericin B, which were diluted to 1X using sterile water. All cells were maintained at 37°C in an atmosphere of 5% CO_2_ with 95% humidity.

Cells were cultured in 96-well plates (Guangzhou Jet Bio-Filtration Products, Co., Ltd., Guangzhou, China) at a density of 2×10^5^ cells/well for 24 h. Following incubation the medium was changed and the cells in the 100-μl medium were treated with 50 μl of various 10-fold serial concentrations of PTA along with the negative control, 0.1% Triton^TM^ X-100 in phosphate-buffered saline (Sigma-Aldrich); the 100-μl medium alone served as the positive control. In addition, the content of the PTA standard was analyzed by a high-performance liquid chromatography system consisting of a Waters model 515 solvent delivery system, a Waters model 996 Photodiode-array detector and a Waters 717 plus autosampler (Waters Corporation, Milford, MA, USA). The concentrations of PTA in the wells were 500, 50, 5, 0.5, 0.05, 0.005, and 0.0005 μg/ml and the cells were incubated for 24 h. MTT and LDH assays were performed promptly following incubation. Three replicates were performed in the same plate and all of the experiments were repeated four times.

### MTT analysis

Following the 24-h cultivation of cells, the media was removed and 10 μl MTT solution (0.5 mg/ml in phosphate-buffered saline) was added. Following incubation (at 37°C for 4 h), formazan crystals were dissolved in 1% sodium dodecyl sulfate (100 μl). The cell viability was subsequently quantified using a microplate reader (Sunrise™; Tecan, Männedorf, Switzerland) at a wavelength of 540 nm (15,16).

### LDH analysis

LDH concentration in the media was measured using a TML Test kit (TR90321/LDH-P 4+1; Tanı Medikal, Ankara, Turkey), which enabled the spectrophotometric determination of the nicotinamide adenine dinucleotide reduction at a wavelength of 340 nm in the presence of lactate and LDH, according to the manufacturer’s instructions. Controls were established with 0.1% (w/v) Triton^TM^ X-100. The relative LDH release was calculated using the following formula: Relative LDH release = ratio of LDH released/total LDH in the intact cells (17,18).

Cytotoxicity was calculated with regards to the untreated cell control, which was set to 100% viability (maximal viability). The dead cell control (Triton^TM^ X-100) was set to 0% viability (minimal viability). The degree of cytotoxicity of PTA-treated cells was expressed as a percentage of the untreated cell control. A plot of percentage cytotoxicity versus sample concentrations was used to calculate the concentrations that exhibited 50% cytotoxicity (termed the half maximal inhibitory concentration; IC_50_) (19).

### Statistical analysis

Measured data were plotted against the corresponding inhibition values, generating inhibition curves for regression analysis, selected by the highest correlation coefficient (R^2^). IC_50_ values were calculated by interpolation of the experimental data using prediction outcomes. The differences between the cytotoxicity measurements of the cell lines (CrFK and HGC-27) and methods (LDH and MTT) were evaluated by Student’s t-test, following data normalization using the Shapiro-Wilk test and the parametric testing of the homogeneity of variances using Levene’s test. The correlation between the cytotoxicity levels obtained by the LDH and MTT methods for each cell line (CrFK and HGC-27) were determined by the Pearson correlation test. All data were analyzed using SPSS version 14.01(SPSS, Inc., Chicago, IL, USA) and P<0.05 was considered to indicate a statistically significant difference for all comparisons.

## Results

### Percentage cytotoxicity

**Figure 1:**
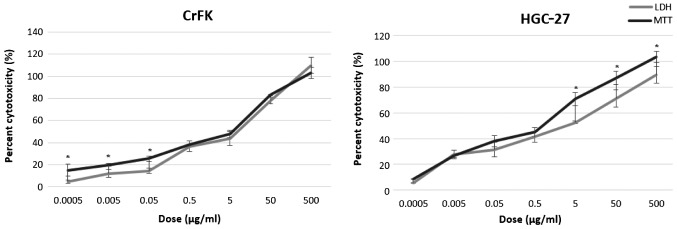
Percentage cytotoxicity of ptaquiloside on CrFK and HGC-27 cells was analyzed using LDH and MTT assays. CrFK, Crandall feline kidney; LDH, lactose dehydrogenase; MTT, 3-(4,5-dimethylthiazol-2-yl)-2,5-diphenyltetrazolium bromide.

**Figure 2:**
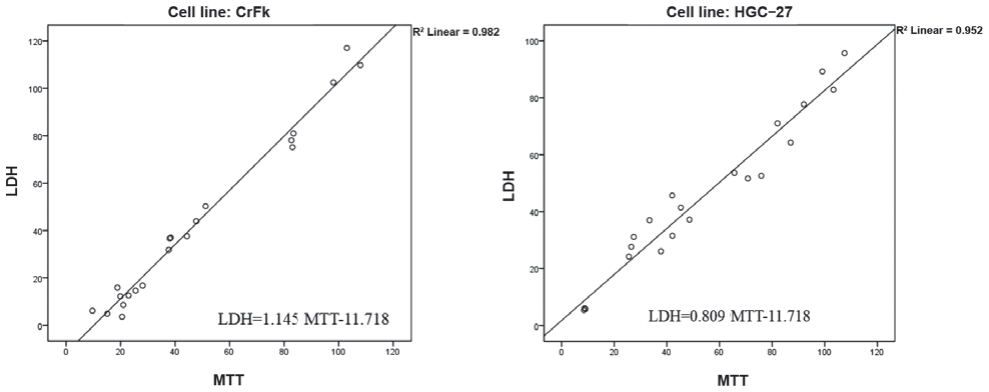
Correlation between the cytotoxicity levels of LDH and MTT assays for each cell line. LDH, lactose dehydrogenase; MTT, 3-(4,5-dimethylthiazol-2-yl)-2,5-diphenyltetrazolium bromide; CrFK, Crandall feline kidney; R^2^, correlation coefficient.

The percentage cytotoxicity increased in the CrFK and HGC-27 cells in a dose-dependent manner following 24 h of PTA exposure (Table I; Fig. 1). IC_50_ values calculated for CrFK cells were 11.17 and 11.86 μg/ml, and were 2.03 and 2.56 μg/ml for the HGC-27 cells, using the MTT and LDH assays, respectively (Table II). These results indicate that gastric HGC-27 cells are more sensitive to PTA than the CrFK cells. At each PTA dose (0.005, 0.005, 0.5, 5, 50 and 500 μg/ml) the differences in percentage cytotoxicity were found to be statistically significant (with the exception of the MTT results at 0.005 μg/ml). Furthermore, the results of the LDH and MTT assays were also found to positively correlate (P<0.001; Fig. 2).

## Discussion

Bracken fern has been categorized as a member of the 2B group of carcinogens by the International Agency for Research on Cancer (IARC; 1998) (20). The 2B group includes agents, mixtures and exposure circumstances for which there is limited evidence of carcinogenicity in humans; however, sufficient evidence of carcinogenicity in experimental animals has been obtained (20).

Few studies have investigated the acute cytotoxic effects of PTA and other associated compounds *in vitro*. Matsuoka *et al* (12) investigated the mutagenicity of PTA and its associated compounds, hypoloside B and C, and illudin M and S, on a Chinese hamster lung cell line using chromosomal aberration tests. PTA induced chromosomal aberrations at doses as low as 4.5 μg/ml (0.0113 mM) and the clastogenic effect was identified to be pH-dependent. Furthermore, hypoloside B and C, and illudin M and S were found to be clastogenic and associated with the carcinogenic potency of PTA in animals. Mori *et al* (21) investigated the genotoxicity of PTA on primary cultures of hepatocytes using DNA-repair tests, where it elicited evident unscheduled DNA synthesis with a dose-response effect. In addition, Ngomuo and Jones (22) evaluated the cytotoxic effects of quercetin, shikimate, cyclohexane-carboxylate and PTA on Chinese hamster ovary mouse fibroblast (3T3) and normal rat kidney cells, and the inhibitory concentrations were found to be high (IC_50_, 0.6×10^−1^ M for all cell lines) when compared with the positive control, mitomycin C (IC_50_, 1×10^−6^ M). Therefore, this low cytotoxic activity was not considered to be directly associated with the etiological agents of acute cattle bracken fern poisoning.

Gomes *et al* (9) investigated the toxic effects of *P. aquilinum* extracts, and the PTA toxin in AGS and MKN-45 gastric epithelial cell lines. The study compared the damaging effects at the cellular and DNA level in gastric cells *in vitro* and in a mouse model. γH2AX and p53-binding protein 1 analysis identified the induction of DNA strand breaks in treated cells. Additionally, p53 levels were increased following exposure, which was associated with ataxia telangiectasia and Rad3-related checkpoint kinase 1 signaling pathway activation. Furthermore, even at a particularly low dose of PTA (10 μg/ml), a decrease in cell viability was observed in the two cell lines, whereas, following 24 and 48 h of exposure to 60 μg/ml PTA, only a marginal increase in the number of late apoptotic and necrotic cells was observed. It was also revealed that MKN-45 cells were less sensitive to the toxic effects of the PTA toxin compared with AGS cells (9). Furthermore, Takaishi *et al* (23), found that MKN-45 cells have a significant side population fraction, which may be associated with tumorigenic ability *in vitro* and *in vivo*. In xenograft models, the MKN-45 cell line exhibited a high proportion of cell surface marker cluster of differentiation (CD)44(+) cells, which are highly tumorigenic in xenograft models and are capable of spheroid colony formation, however, AGS cells did not exhibit a measurable side population fraction. Side population cells express high levels of various members of the ATP-binding cassette transporters family, which are responsible for drug resistance; thus, blocking these transporters presents a potential target for cancer therapy (24). The decreased sensitivity of MKN-45 cells to PTA cytotoxicity may be attributed to the high expression of CD44 and surface proteins, which are associated with its carcinogenic effects and decreased cytotoxicity. Song *et al* (25) investigated the association between the sonic hedgehog signaling pathway and gastric cancer stem cells, where CD44 expression was found to be higher in MKN-45 cells than in HGC-27 cells, in tumorsphere-derived cells. In the current study, a more aggressive tumorigenic cell line, HGC-27, cultured from the metastatic lymph node of a gastric cancer patient histologically diagnosed with undifferentiated carcinoma was used, which resulted in a higher sensitivity with regards to cell viability when compared with MKN-45 cells that were cultured from a poorly differentiated adenocarcinoma of the stomach.

van den Bout-van den Beukel *et al* (26) investigated the cytotoxic, genotoxic and cytochrome P450 (CYP) enzymatic competition effects of Tanzanian plant extracts traditionally administered for the treatment of fungal infections, including *Pteridium aquilinum*. Methanolic extracts were applied to HepG2 and HeLa cells and the mitochondrial activity, cellular proliferation, damage to the cellular membrane, glutathione depletion and electron transport chain activity were investigated using Alamar Blue, Hoechst 33342, calcein-AM uptake, glutathione depletion and O_2_-consumption assays. *P. aquilinum* did not reduce the nicotinamide adenine dinucleotide phosphate-oxidase content, exhibited no effect on the mitochondrial activity with Alamar Blue assay and did not result in a significant reduction in DNA levels with Hoechst 33342 at concentrations of ≤500 μg/ml. IC_50_ values of the plant extract on CYP2C9, 2C19 and 2D6, 3A4 supersome and 3A4 supersome dibenzylfluorescein enzymes were 4.98, 12.06, 70.5, 8.05 and 16.8 μg/ml, respectively, exhibiting the highest inhibition on the CYP2C9 enzymes. No studies regarding the CYP expression of the investigated cell lines have been performed, and thus, the underlying cytotoxicity mechanism can not be directly associated with the inhibition of these enzymes.

Campos-da-Paz *et al* (13) investigated the effects of vitamin C (10 and 100 μg/ml) on the reversibility of DNA damage caused by bracken on human submandibular gland (HSG) and oral epithelium cells (OSCC-3), which had previously been exposed to bracken-fern extract. The results demonstrated that bracken-fern extract (31 mg/ml) was cytotoxic to HSG and OSCC-3 cells, causing cell death by apoptosis, and that vitamin C was not able to reverse these effects.

Chen *et al* (14) isolated three novel compounds from the ethyl acetate extract of *Pteris ensiformis*, which were investigated for cytotoxicity in the Hep G2 (human liver cancer), A549 (human lung carcinoma), MDA-MB-231 (breast carcinoma), MCF-7 (breast carcinoma), Ca9-22 (Human gingival carcinoma cell line) and HL 60 (human leukemia) cell lines using the MTT assay. Among them, an isolated compound, β-D-xylopyranosyl(1→2)-7-O-benzoyl-β-D-glucopyranoside and pterosin B exhibited selective activity against HL 60 cells, with IC_50_ values of 3.7 and 8.7 μg/ml, respectively. Furthermore, the illudin-series compounds were identified as precursors of pterosins. Pterosins are inactive in antioxidant assays; however, they perform a critical cytotoxic role in cancer cell lines. Although PTA is converted to pterosin B *in vitro*, it exhibits highly cytotoxic properties.

In conclusion, the results from the current study demonstrate the antiproliferative activities of PTA. The underlying mechanisms of this bracken toxin in exhibiting anti-gastric cancer activities may only occur in cancer cell lines. However, the efficacy of PTA on the inhibition of gastric cancer cell growth *in vitro* at relatively low concentrations indicates its cytotoxic effect in healthy cells. Thus, the analyses presented in the current study provide significant evidence for the safety and efficacy of PTA.

## Figures and Tables

**Table I:** Percentage cytotoxicity of PTA in CrFK and HGC-27 cells analyzed by LDH and MTT assays.

	CrFK			HGC-27		
		
PTA (μg/ml)	LDH (%)	MTT (%)	P-value	LDH (%)	MTT (%)	P-value
0.0005	4.82±1.28	15.10±5.44	<0.05	5.76±0.33	8.76±0.29	<0.05
0.005	12.20±3.66	19.91±1.08	<0.05	27.67±3.45	26.48±0.93	NS
0.05	14.63±2.11	25.50±2.57	<0.01	31.53±5.46	37.75±4.35	NS
0.5	36.59±4.79	38.03±0.36	NS	41.42±4.23	45.30±3.28	NS
5	43.90±6.33	47.76±3.42	NS	52.61±0.97	70.77±5.11	<0.01
50	78.05±2.90	83.05±0.38	NS	70.99±6.71	87.04±5.00	<0.05
500	109.76±7.25	103.02±4.97	NS	89.24±6.40	103.31±4.22	<0.01

PTA, ptaquiloside; CrFK, Crandall feline kidney; LDH, lactose dehydrogenase; MTT, 3-(4,5-dimethylthiazol-2-yl)-2,5-diphenyltetrazolium bromide; NS, not significant.

**Table II:** IC_50_ values of PTA on CrFK and HGC-27 cells analyzed by LDH and MTT assays.

	IC_50_ (μg/ml)	
	
Analysis method	CrFK	HGC-27
LDH	11.86	2.56
MTT	11.17	2.03

IC_50_, half maximal inhibitory concentration; PTA, ptaquiloside; CrFK, Crandall feline kidney; LDH, lactose dehydrogenase; MTT, 3-(4,5-dimethylthiazol-2-yl)-2,5-diphenyltetrazolium bromide.

## References

[1] Pamukcu AM, Price JM, Bryan GT (1976). Naturally occurring and bracken-fern-induced bovine urinary bladder tumors. Clinical and morphological characteristics. Vet Pathol.

[2] Peioxoto PV, Nascimento França T, Barros CSL, Tokarina CH (2003). Histopathological aspects of bovine enzootic hematuria in Brazil. Pesq Vet Bras.

[3] Meuten DJ, Everitt J, Inskeep W, Jacobs RM, Peleteiro M, Thompson KJ, Schulman FY (2004). Urinary bladder tumours. WHO Histological Classification of Tumours of the Urinary System of Domestic Animals.

[4] Kushida T, Uesugi M, Sugiura Y (1994). DNA damage by ptaquiloside, a potent bracken carcinogen: detection of selective strand breaks and identification of DNA cleavage products. J Am Chem Soc.

[5] Shahin M, Moore MR, Worrall S, Smith BL, Seawright AA, Prakash AS (1998). H-ras activation is an early event in the ptaquiloside-induced carcinogenesis: comparison of acute and chronic toxicity in rats. Biochem Biophys Res Commun.

[6] Carvalho T, Naydan D, Nunes T, Pinto C, Peleteiro MC (2009). Immunohistochemical evaluation of vascular urinary bladder tumors from cows with enzootic hematuria. Vet Pathol.

[7] Alonso-Amelot ME, Castillo U, De Jongh F (1993). Passage of the bracken fern carcinogen ptaquiloside into bovine milk. Lait.

[8] Alonso-Amelot ME, Castillo U, Smith BL, Lauren DR (1998). Excretion, through milk, of ptaquiloside in bracken-fed cows. A quantitative assessment. Lait.

[9] Gomes J, Magalhães A, Michel V (2012). Pteridium aquilinum and its ptaquiloside toxin induce DNA damage response in gastric epithelial cells, a link with gastric carcinogenesis. Toxicol Sci.

[10] Bonadies F, Borzacchiello G, Dezzi S, Nicoletti R, Roperto S (2004). Mass spectrometric analysis of ptaquiloside, the toxic sesquiterpene from bracken fern. Rapid Commun Mass Spectrom.

[11] Galpin OP, Whitaker CJ, Whitaker R, Kassab JY (1990). Gastric cancer in Gwynedd. Possible links with bracken. Br J Cancer.

[12] Matsuoka A, Hirosawa A, Natori S, Iwasaki S, Sofuni T, Ishidate M (1989). Mutagenicity of ptaquiloside, the carcinogen in bracken, and its related illudane-type sesquiterpenes. II Chromosomal aberration tests with cultured mammalian cells. Mutat Res.

[13] Campos-da-Paz M, Pereira LO, Bicalho LS, Dórea JG, Poças-Fonseca MJ, de Santos MF (2008). Interaction of bracken-fern extract with vitamin C in human submandibular gland and oral epithelium cell lines. Mutat Res.

[14] Chen YH, Chang FR, Lu MC, Hsieh PW, Wu MJ, Du YC, Wu YC (2008). New benzoyl glucosides and cytotoxic pterosin sesquiterpenes from *Pteris ensiformis* Burm. Molecules.

[15] Mossman T (1983). Rapid colorimetric assay for cellular growth and survival: application to proliferation and cytotoxicity assays. J Immunol Methods.

[16] Horáková K, Sovcíková A, Seemannová Z, Syrová D, Busányová K, Drobná Z, Ferencík M (2001). Detection of drug-induced, superoxide-mediated cell damage and its prevention by antioxidants. Free Radic Biol Med.

[17] Butler M (2004). Viability measurements. Animal Cell Culture and Technology.

[18] Fotakis G, Timbrell JA (2006). In vitro cytotoxicity assays: comparison of LDH, neutral red, MTT and protein assay in hepatoma cell lines following exposure to cadmium chloride. Toxicol Lett.

[19] Ulukaya E, Ozdikicioglu F, Oral AY, Demirci M (2008). The MTT assay yields a relatively lower result of growth inhibition than the ATP assay depending on the chemotherapeutic drugs tested. Toxicol In Vitro.

[20] IARC Working Group (1987). Bracken fern (*Pteridium aquilinum*) and some of its constituents. IARC Monographs on the Evaluation of Carcinogenic Risks to Humans.

[21] Mori H, Sugie S, Hirono I, Yamada K, Niwa H, Ojika M (1985). Genotoxicity of ptaquiloside, a bracken carcinogen, in the hepatocyte primary culture/DNA-repair test. Mutat Res.

[22] Ngomuo AJ, Jones RS (1996). Cytotoxicity studies of quercetin, shikimate, cyclohexanecarboxylate and ptaquiloside. Vet Hum Toxicol.

[23] Takaishi S, Okumura T, Tu S (2009). Identification of gastric cancer stem cells using the cell surface marker CD44. Stem Cells.

[24] Hadnagy A, Gaboury L, Beaulieu R, Balicki D (2006). SP analysis may be used to identify cancer stem cell populations. Exp Cell Res.

[25] Song Z, Yue W, Wei B (2011). Sonic hedgehog pathway is essential for maintenance of cancer stem-like cells in human gastric cancer. PLoS One.

[26] van den Bout-van den Beukel CJ, Hamza OJ, Moshi MJ (2008). Evaluation of cytotoxic, genotoxic and CYP450 enzymatic competition effects of Tanzanian plant extracts traditionally used for treatment of fungal infections. Basic Clin Pharmacol Toxicol.

